# A comprehensive interventional program for promoting eating behaviors in adolescent girls with polycystic ovarian syndrome (PCOS): protocol for a mixed methods study

**DOI:** 10.1186/s12978-018-0652-y

**Published:** 2018-12-04

**Authors:** Leila Hajivandi, Mahnaz Noroozi, Firoozeh Mostafavi, Maryam Ekramzadeh

**Affiliations:** 1 Faculty of Nursing and Midwifery, Isfahan University of Medical Sciences, Isfahan, Iran; 2 Department of Midwifery and Reproductive Health, School of Nursing and Midwifery, Isfahan University of Medical Sciences, Isfahan, Iran; 3 Department of Health Education and Promotion, School of Health, Isfahan University of Medical Sciences, Isfahan, Iran; 4 Department of Clinical Nutrition, School of Nutrition and Food Sciences, Shiraz University of Medical Sciences, Shiraz, Iran

**Keywords:** Eating behaviors, Polycystic ovarian syndrome, Adolescent, Mixed methods study, Iran

## Abstract

**Background:**

Polycystic ovarian syndrome (PCOS) is the most common endocrine disorder among women. Inappropriate eating behaviors are some of the most important risk factors for obesity in all age groups. Therefore, performing comprehensive culturally sensitive interventions for modification of eating behaviors as a useful affordable strategy seems necessary. So, this study aims to present a comprehensive interventional program for promoting eating behaviors in adolescent girls with PCOS.

**Methods:**

This study has a sequential exploratory mixed methods design including three sequential phases. The researcher represents eating behaviors among adolescent girls with PCOS using a qualitative approach. In the onset of the second phase, a comprehensive interventional program for promoting eating behaviors is designed for adolescent girls with PCOS. In this regard, in addition to qualitative studies, some related papers and texts are used. The suggested program of expert panel is approved based on prioritization guidelines. Then, in the third phase and after different stages of finalization of the program, its affectability is evaluated regarding improvement of eating behaviors in adolescent girls with PCOS.

**Discussion:**

Results of the present mixed methods study, by presenting an interventional culturally sensitive program for promoting eating behaviors in adolescent girls with PCOS, lead to the improvements of the health of young girls. If this program works, it can become one of the leading education guidelines for eating behaviors in adolescent girls with PCOS.

**Trial registration:**

IRCT20160224026756N6. Registered 18 Aug 2018.

## Plain English summary

Polycystic ovarian syndrome (PCOS) is a kind of hormonal imbalance among women of reproductive age. It would be indicated by symptoms such as obesity, hirsutism, menstrual cycle disorder and infertility, alone or together. In women and young girls with this syndrome, obesity is one of the pathophysiological principles; consequently, increased level of insulin in these patients might stimulate the production of ovarian androgens and hence, would lead to ovulation disorder and infertility. Inappropriate eating behaviors are one of the main causes of obesity. Since overweight and obesity have a principal role in causing, development of and complications caused by PCOS, appropriate weight and having healthy eating behaviors might significantly decrease the complications of PCOS among adolescent girls. The findings of this study are suitable sources for selecting the best intervention for promoting eating behaviors in adolescent girls with PCOS. This study is carried out by an exploratory sequential mixed qualitative-quantitative methods approach including three sequential phases. In this study, the researcher explains eating behaviors in adolescent girls with PCOS using a qualitative approach. In the onset of the second phase, a comprehensive interventional program is designed for promoting eating behaviors in these adolescent girls. In addition to qualitative study, some related papers and texts are studied. The suggested interventional program of expert panel is approved and validated based on prioritization guidelines. In the third phase and after different stages of finalization of the program, its affectability is evaluated regarding improvement of eating behaviors in adolescent girls with PCOS. It is expected that using the results of the present study and applying its suggested interventional program by adolescent girls, policymakers, health managements and families might have positive effects on administering and promoting healthy eating behaviors by adolescent girls with PCOS.

## Background

Polycystic ovarian syndrome (PCOS) was first explained 70 years ago [1, 2]. This syndrome is a kind of hormonal imbalance among women of reproductive age which its prevalence among the adults, according to the criteria by American National Institute, has been reported as 6.5%. PCOS is a heterogeneous disease; at one end of its spectrum, it shows morphological polycystic ovary symptoms in the ultrasound examination, and at the other end of the spectrum, it would be indicated by symptoms such as obesity, hyperandrogenism, menstrual cycle disorder and infertility, alone or together. In women and young girls with this syndrome, obesity is one of the pathophysiological principles; consequently, increased level of insulin in these patients might stimulate the production of ovarian androgens and hence, would lead to ovulation disorder and infertility [3, 4]. Obesity could aggravate all of the infertility and metabolic problems of this syndrome [5, 6]. According to string evidences, PCOS would onset during adolescence but, unlike the adults, its diagnosis is difficult among the adolescents [7]. The prevalence of this disease among 11–19 years old girls, based on diagnostic criteria, has been reported as 1.8–15% [8].

The global prevalence of obesity among adolescents is 12 to 20% [9]. Inappropriate eating behaviors are one of the main causes of obesity [10, 11]. Nutritional habits usually form during adolescence and continue to adulthood, so they could affect eating behaviors and the intake of the nutrients. Since overweight and obesity have a principal role in causing, development of and complications caused by PCOS, according to studies, appropriate weight and having healthy eating behaviors might significantly decrease the complications of PCOS among adolescent girls [6]. So, weight loss and lifestyle modification are defined as the first line treatments for this syndrome [12]. So, various low-calorie diets with different combinations, including macronutrients, have been suggested for weight loss of obese people. But the existing information about the condition of adolescents with PCOS and their nutritional behaviors is so limited [13] and no studies have been conducted in this field in Iran. Considering the long-term and life-threatening complications of PCOS, including metabolic, cardiovascular and especially infertility, and also imposing heavy costs on individuals and the society, the need for modification of nutritional behaviors, as a useful affordable strategy, seems necessary. Therefore, the aim of the present study is to present a comprehensive interventional program for promoting eating behaviors in adolescent girls with PCOS.

### Objectives

Objectives of each phase are as the following:

### Objectives of the first phase: Qualitative study

Explaining eating behaviors in adolescent girls with PCOS.

### Objectives of the second phase: Program design

Designing a primary intervention program based on extracted data from qualitative phase and review.

### Objectives of the third phase: Quantitative study

Explaining affectability of the comprehensive interventional program for promoting eating behaviors in adolescent girls with PCOS.

## Methods/ design

**Fig. 1:**
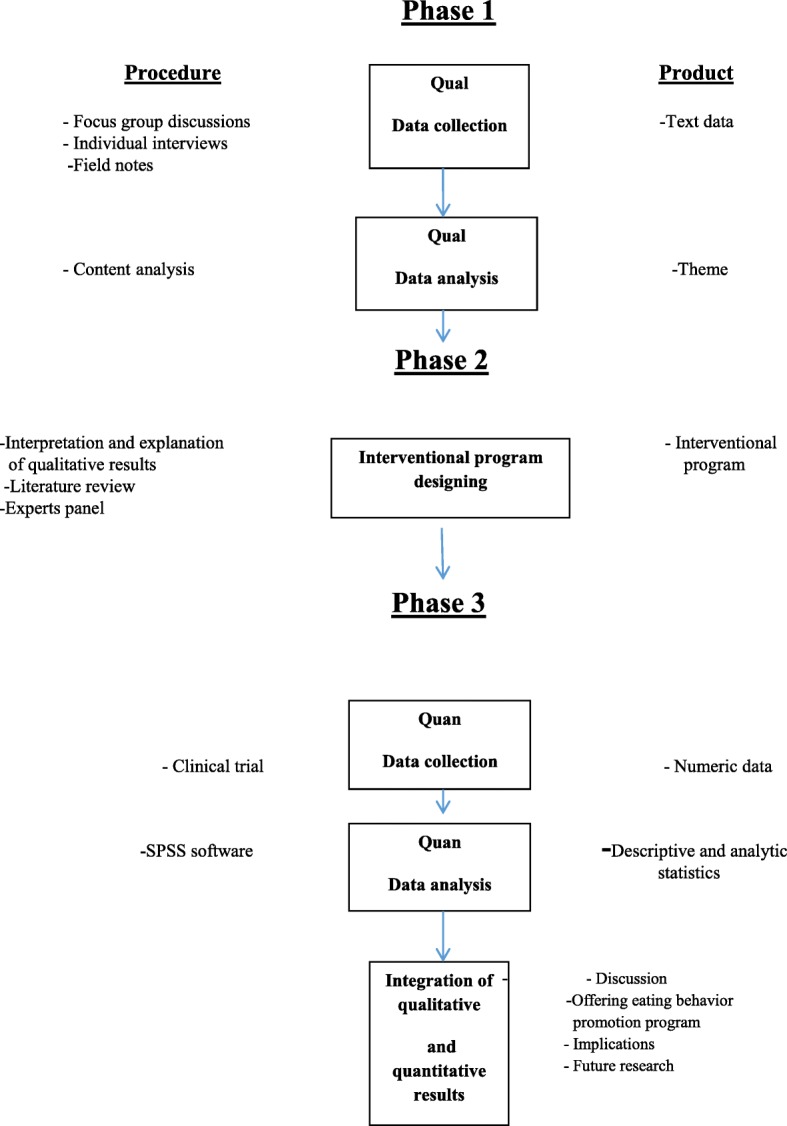
Study visual diagram

The present study is a mixed method interventional study with a sequential design (qualitative-quantitative) including three sequential phases. The researchers use a qualitative approach to explain eating behaviors in adolescent girls with PCOS and the required strategies for their healthy nutritional behavior. For this end, they analyze experiences of the participants. In this phase, the data are collected via semi-structured individual in-depth interviews, focused group discussions, and field noting. The qualified participants are selected with a purposeful approach and with maximum variety. Simultaneous with data collection, the interviews will be analyzed with a conventional content analysis method. Sampling and coding will be continued until saturation occured. After saturation and at the end of interviews, the researcher enters the second phase of the study in which a comprehensive program will be designed for promoting eating behaviors in adolescent girls with PCOS. For this end, a literature review of papers and texts will be used in addition to the results of qualitative study. Then, based on the results of the qualitative study, literature review and holding expert panel, a comprehensive program will be designed for modification of healthy eating behaviors among adolescent girls with PCOS. Then, the suggested program will be validated based on prioritization guidelines. In the third phase and after different stages of finalization of the program, its affectability is evaluated regarding improvement eating behaviors in adolescent girls with PCOS. The collected data will be processed by SPSS Version 20.0 software and will be analyzed with descriptive-analytic statistical methods (Fig. 1).

### First phase: Qualitative study

The first phase of this study is designed for answering the question of: “What do you eat during the day” from adolescent girls with PCOS or “Do usually adolescent girls with PCOS have problems in the food dietary?” from the viewpoint of other participants. This study will be carried out using a qualitative content analysis method.

### Participants in the qualitative phase

Research community of the first phase consists of adolescent girls with PCOS and their parents. Other participants of this phase of the study will be midwives, gynecologists, endocrinologists and dietitians and also authorities of the adolescents and youth health unit of the health centers of Shiraz, Iran.

### Sampling method

In this phase of study, participants are selected with a purposeful sampling method. They are selected based on maximum variety in age, education, job, marriage status and duration of the diagnosis of disease.

### Inclusion criteria for participants

- Being an adolescent girl from 15 to 21 years old (for diagnosis and definition of PCOS, at least two years must be passes from menarche) [14].
- Willingness to participate in the study and obtaining informed consent from the adolescent and her parents (in cases of younger than 18 years old)
- Overweight or obese adolescent girls with PCOS that their diagnosis of PCOS has been confirmed by a gynecologist
- Not having a history of diagnosed major mental problems undergoing drug treatments
- Not having any chronic diseases (cardiovascular diseases, diabetes, renal diseases, etc.), which requires special diet prescribed by a physician

### Research environment

The participants are accessed through the health centers and hospitals covered by Shiraz University of Medical Sciences and gynecologists and midwives offices. The interviews will be done in the location and time selected by the participants for their comfort and ease.

### Data collection process

After obtaining permission from Shiraz University of Medical Sciences, the researchers will select the participants by referring to research environments. In the qualitative phase, data collection methods include individual, in-depth, open, and semi-structured interviews along with focused group discussions and field noting. The interviews will be recorded. After explaining objectives and methodology of the study, the researcher will receive written consent regarding participation in the research, further interviews, and recording the interviews. Location of the interviews will be selected by the participants. In semi-structured individual interviews, the first interviews are done with the aim of understanding possible and unpredictable issues. The general questions of semi-structured interviews are identified based on the resulted information. At the end of each interview, the narrative will be transcribed immediately, and data analysis will be done simultaneously with data collection. Data collection continues until saturation - as long as no new code or data is extracted. At this point, data saturation and adequacy will be verified.

### Data analysis

A conventional content analysis method, introduced by Graneheim & Lundman (2004), is used for data analysis [15]. Each interview will be transcribed immediately at the end of recording. After extracting the general idea, the narrative will be analyzed line by line, and its meaning units will be identified. Compact meaning units and codes will be extracted from these meaning units. After extracting the primary code, the data will be reduced and divided into sub- categories and main categories, based on their appearance.

### Rigor and trustworthiness

For reliability and validity analysis, four criteria are suggested: credibility, dependability, transferability, and confirmability [16]. Different measures will be taken into consideration in order to improve credibility of this study, such as selecting the participants with maximum variety, spending sufficient amount of time on data collection, performing in-depth interviews in different locations and times, and mixing multiple data collection methods including individual interviews, focused group discussions, and field noting. For verification of extracted codes and data or their modification, they will be reviewed by the participants.

For confirming the reliability of the findings, some examples of code extraction methods and their corresponding interview narratives will be reviewed by an external supervisor in order to control the accuracy of researcher’s perception and to find contradictory cases. For increasing transferability, study findings will be presented to people who have similar characteristics with the participants in order to compare the results of this study with their own experiences. Regarding verification, the researcher will explain the whole procedure, including recording, transcription, code extraction, and categorization. In order to verify the coding procedure, some of the research colleagues and faculty members, who are acquainted with qualitative research analysis and do not want to participate in this research, are asked to review the procedure.

### Second phase: Designing the intervention program

After collecting the required data by qualitative study, the second phase of the study starts. The purpose of this phase is designing an intervention program for improving eating behavior in adolescent girls with PCOS. In this phase, the required strategies are extracted based on the results of qualitative phase and a literature review of papers and texts. These strategies will be validated by expert panel. The method of review will be narrative review with searching in electronic and library resources including reference books and theses. Multiple databases are available for searching the related papers, such as Scopus, MEDLINE, Ovid, ProQuest, Cochrane Library, Science Direct, Web of Science, PubMed, Embase, Magiran, SID, Google Scholar and CINAHL.

In this phase, all the English and Persian qualitative, quantitative, and mixed methods studies on eating behaviors, eating problems and healthy eating behavior facilitators in adolescent girls with PCOS, which have been published during 1991 to 2018, will be studied and analyzed. In the next phases, search will be narrowed down to specific mixed keywords, including “healthy eating behaviors”, eating behaviors in adolescent girls with PCOS and “eating problems”, eating behaviors in adolescent girls with PCOS and “nutritional health needs”, eating behaviors in adolescent girls with PCOS and “nutritional health promotion”, eating behaviors in adolescent girls with PCOS and “weight management”, eating behaviors in adolescent girls with PCOS and “lifestyle and behavioral management”, eating behaviors in adolescent girls with PCOS and “elimination of eating problems”, eating behaviors in adolescent girls with PCOS and “nutritional management”, eating behaviors in adolescent girls with PCOS and “life style modification”, eating behaviors in adolescent girls with PCOS and “nutritional intervention”.

### Holding a panel of experts

The objectives of this phase is to extract relevant eating strategies for having healthy eating behaviors in adolescent girls with PCOS from qualitative study and analyze the interview narratives in expert panel in order to prioritize their function. Then, the suitable interventional program will be selected and executed in the quantitative phase based on this prioritization. For this end, a decision making matrix will be extracted for prioritization of the extracted strategies from qualitative study and literature review of papers and texts. In this matrix, each strategy is provided with a score from one to three based on three criteria of costs, ease of execution, and time. This matrix will be sent for some specialists in the Delphi Round 1, average score will be calculated for each solution, high priority strategies will be identified, and consequently, a suitable intervention method will be selected. In Delphi Round 2, the selected intervention method will be evaluated qualitatively in a meeting with the presence of research team and panel members (nutrition and food sciences specialists, psychologists, gynecologists, reproductive health professionals, endocrinologist and authorities of the adolescents and youth health). A copy of the intervention strategy will be presented to the experts who will be returned to the researcher at the end of the meeting. Comments and suggestions will be collected and applied in order to finalize and execute the strategy in the third phase (quantitative study).

### Third phase: Quantitative study

#### Type of quantitative study

Quantitative phase of the study will be carried out using a two-group clinical trial.

#### Research population

The targeted population for quantitative study is all adolescent girls with PCOS referred to gynecology training clinics covered by Shiraz University of Medical Sciences.

#### Research sample

Study sample includes adolescent girls with PCOS who will be selected by convenience sampling and have all the inclusion criteria.

#### Research environment

This study will be carried out in four gynecology training clinics covered by Shiraz University of Medical Sciences. The reason for selecting such kind of environment is easy access to adolescent girls with PCOS and characteristics of the research unit.

#### Sample size

Sample size will be 36 participants in each group considering 95% confidence interval, 80% trial power, S = 0.1–0.25 and 10% loss.

#### Sampling method

This clinical trial has one intervention group and one control group. After identifying the clinics covered by Shiraz University of Medical Sciences, four gynecology training clinics will be selected non-randomly. Afterwards, 18 adolescent girls with PCOS with inclusion criteria will be selected from each center via convenience sampling method.

#### Inclusion criteria

- Being an adolescent girl from 15 to 21 years
- Single, overweight or obese adolescent girls with PCOS that their diagnosis of PCOS has been confirmed by a gynecologist
- Willingness to participate in the study and obtaining informed consent from the adolescent and her parents (in cases of younger than 18 years old)
- Not having a history of diagnosed major mental problems undergoing drug treatments
- Not having any chronic diseases (cardiovascular diseases, diabetes, renal diseases, etc.), which requires special diet prescribed by a physician
- Not attending a clinical trial simultaneously

#### Exclusion criteria

- Unwillingness to continue cooperation during the trial
- Use of certain dietary patterns during study
- Failure to receive 50% of the intervention for any reason

#### Research variables

In this clinical trial, the designed interventions are considered to be independent variables and.

knowledge, attitude, subjective norms, perceived behavioral control, behavioral intention, and eating practice (behavior) in adolescent girls with PCOS are considered to be dependent variables.

#### Data collection methods

Researcher-made questionnaires evaluating knowledge, attitude, subjective norms, perceived behavioral control, behavioral intention and eating practice (behavior) in adolescent girls with PCOS are used in the quantitative phase of this trial. Reliability and validity of these questionnaires will be determined. Knowledge questionnaire includes 11 questions with three options of “true, false and I do not know”. In measuring knowledge score, one score will be given to the participants for each true answer. Minimum and maximum knowledge scores are 0 and 11, respectively. Attitude questionnaire includes nine questions with a five-point Likert scale of “strongly disagree, disagree, neither agree nor disagree, agree, and strongly agree”. Scoring is done from one to five. Minimum and maximum attitude scores are nine and 45, respectively. Subjective norms questionnaire includes six questions with five options of “never true, rarely true, sometimes but infrequently true, and always true”. In measuring subjective norms score, one score will be given to the participants for each true answer. Minimum and maximum subjective norms scores are six and 30, respectively. Perceived behavioral control questionnaire includes seven questions with a five-point Likert scale of “strongly disagree, disagree, neither agree nor disagree, agree, and strongly agree”. Scoring is done from one to five. Minimum and maximum perceived behavioral control scores are seven and 35, respectively. Behavioral intention questionnaire includes eight questions with five options of “certainly, I do this; most likely, I do this; it is possible, I do this; Unlikely, I do this; and never I do this”. In measuring behavioral intention score, one score will be given to the participants for each true answer. Minimum and maximum behavioral intention scores are eight and 40, respectively. Food frequency questionnaire (FFQ) is used for evaluation eating practice (behavior). Participants were asked to indicate, on average, the frequency of each food item (out of 22) they consumed during the previous one week. The frequency was asked in four categories ranging from ‘never’ to ‘daily’.

#### The implementation method

The researcher will implement the designed program after obtaining the necessary coordination with authorities of four gynecology training clinics covered by Shiraz University of Medical Sciences. This is done by referring to the gynecology training clinics and presenting a letter of introduction to the authorities of those centers regarding research objectives. After that, the researcher will be allowed to access to phone numbers of adolescent girls with PCOS and call her in order to explain research objectives and invite them to participate in the research. If they are willing to cooperate, and have all the inclusion criteria, they will be selected by convenience sampling method with their informed consent.

Adolescent girls^,^ knowledge, attitude, subjective norms, perceived behavioral control, behavioral intention and eating practice (behavior) will be considered as the suggested outcomes. Before intervention, the questionnaires will be filled by them in each of the two groups. Furthermore, the questionnaires will be refilled by adolescent girls with PCOS three months after the intervention.

#### Data analysis

The collected data will be analyzed by descriptive statistical methods (mean, standard deviation, minimum, and maximum), inferential statistics (paired t-test, t-test, Chi-squared test, Fishers exact test, Wilcoxon test, and Mann-Whitney test), and SPSS Version 20.0 software. ANCOVA will be used for adjusting intervening variables.

#### Integration of the qualitative and quantitative data

The results of the qualitative and quantitative phases of the study will be integrated and finally, a program for promoting eating behaviors in adolescent girls with PCOS will be provided.

## Discussion

One of the useful strategies for preventing diet-related chronic diseases is to modify and change eating behaviors. PCOS is one of these diseases that obesity and overweight has an important role in its occurrence, advancement and complications. Also obesity could aggravate all of the problems caused by this syndrome including infertility and metabolic problems [6]. Most of the studies have suggested weight loss and lifestyle modification as the first line treatment for this syndrome [12]. Therefore, it seems necessary to perform fundamental culturally sensitive society-based interventions in this field. In the present study sequential exploratory design will be used for providing an appropriate interventional program. Exploratory mixed method is a known method for performing researches, especially when little information exists about the studied phenomenon; it is also an appropriate method for achieving the experiences of the participants. When one method is not sufficient for revealing the research subject, it is better to use a mixture of both methods. Using mixed method would help the researcher to gain a comprehensive understanding of a phenomenon and would relate different aspects of a phenomenon to each other [17]. Since the subject of the present study has a new multi-dimensional nature and the researcher is not able to predict the effective factors on this subject, therefore it seems that using mixed method study and applying both the methods of qualitative and quantitative studies would be an appropriate method for achieving the goals of the study.

According to the studies, having healthy eating behaviors and a proper weight could significantly decrease the complications of PCOS in adolescent girls [6]. It is believed that improving and enhancing the health condition of the adolescents is much more difficult and complicated that other age groups. Although the mortality rate of the youth and adolescents is relatively low, they are usually involved with behaviors that could affect the quality of their health. However, their behaviors are not rooted and permanent and the ability to accept changes is more among them than the adults. So, the efforts toward the health of the adolescents must mostly consider preventive measures. Furthermore, prevention is a cost-effective strategy for adolescents and its benefits are long-lasting [18]. Therefore, it seems that, using the results of the present study and applying its suggested interventional program by adolescent girls, policymakers, health managements and families might have positive effects on administering and promoting healthy eating behaviors by adolescent girls with PCOS. If this program works, it can become one of the leading education guidelines for eating behaviors in adolescent girls with PCOS.

## Abbreviations

FFQ: Food frequency questionnaire
PCOS: Polycystic ovarian syndrome
